# Plasma glial fibrillary acidic protein detects Alzheimer pathology and predicts future conversion to Alzheimer dementia in patients with mild cognitive impairment

**DOI:** 10.1186/s13195-021-00804-9

**Published:** 2021-03-27

**Authors:** Claudia Cicognola, Shorena Janelidze, Joakim Hertze, Henrik Zetterberg, Kaj Blennow, Niklas Mattsson-Carlgren, Oskar Hansson

**Affiliations:** 1 Clinical Memory Research Unit, Department of Clinical Sciences, Lund University, Lund, Sweden; 2 Memory Clinic, Skåne University Hospital, Malmö, Sweden; 3 Department of Psychiatry and Neurochemistry, Institute of Neuroscience and Physiology, The Sahlgrenska Academy at the University of Gothenburg, Mölndal, Sweden; 4 Clinical Neurochemistry Laboratory, Sahlgrenska University Hospital, Mölndal, Sweden; 5 Department of Neurodegenerative Disease, UCL Institute of Neurology, Queen Square, London, UK; 6 UK Dementia Research Institute at UCL, London, UK; 7 Department of Neurology, Skåne University Hospital, Lund, Sweden; 8 Wallenberg Center for Molecular Medicine, Lund University, Lund, Sweden

**Keywords:** Blood biomarkers, GFAP, Alzheimer’s disease, Mild cognitive impairment

## Abstract

**Introduction:**

Plasma glial fibrillary acidic protein (GFAP) is a marker of astroglial activation and astrocytosis. We assessed the ability of plasma GFAP to detect Alzheimer’s disease (AD) pathology in the form of AD-related amyloid-β (Aβ) pathology and conversion to AD dementia in a mild cognitive impairment (MCI) cohort.

**Method:**

One hundred sixty MCI patients were followed for 4.7 years (average). AD pathology was defined using cerebrospinal fluid (CSF) Aβ42/40 and Aβ42/total tau (T-tau). Plasma GFAP was measured at baseline and follow-up using Simoa technology.

**Results:**

Baseline plasma GFAP could detect abnormal CSF Aβ42/40 and CSF Aβ42/T-tau with an AUC of 0.79 (95% CI 0.72–0.86) and 0.80 (95% CI 0.72–0.86), respectively. When also including *APOE* ε4 status as a predictor, the accuracy of the model to detect abnormal CSF Aβ42/40 status improved (AUC = 0.86, *p* = 0.02). Plasma GFAP predicted subsequent conversion to AD dementia with an AUC of 0.84 (95% CI 0.77–0.91), which was not significantly improved when adding *APOE* ε4 or age as predictors to the model. Longitudinal GFAP slopes for Aβ-positive and MCI who progressed to dementia (AD or other) were significantly steeper than those for Aβ-negative (*p* = 0.007) and stable MCI (*p* < 0.0001), respectively.

**Conclusion:**

Plasma GFAP can detect AD pathology in patients with MCI and predict conversion to AD dementia.

## Introduction

By 2050, more than 150 million people worldwide are estimated to be affected by dementia, with Alzheimer’s disease (AD) causing up to 70% of all cases [1]. AD dementia is still largely a clinical diagnosis, although amyloid-β (Aβ) in brain starts decades before the symptoms appear [2]. Aβ plaques cause astrogliosis, i.e., functional and morphological changes in the surrounding astrocytes, which are glial cells involved in brain signaling, modulation of synapses, transport of nutrients, homeostasis, and structural support [3, 4]. Animal and cell studies have shown the presence of astrogliosis around Aβ plaques and the involvement of reactive astrocytes in Aβ production and toxicity [5–8]. Studies conducted with PET tracers targeting astrocytes have shown that astrogliosis is an early feature in the pathological cascade of AD, which decreases over the course of disease as opposed to the increase of Aβ plaque load [9–11].

Glial fibrillary acidic protein (GFAP) is expressed in the cytoskeleton of astrocytes and has been found significantly increased in CSF in AD and other neurodegenerative diseases compared to healthy controls [12–15]. GFAP has also been recently measured in plasma and serum, where it was found increased in different neurological conditions, including AD [16–24]. Different studies have shown that higher concentrations of plasma GFAP were associated to amyloid-PET positivity and worse outcomes in global cognition [22, 24–27]. Even though previous studies suggest that blood GFAP levels are elevated in AD and can identify an amyloid-PET positive status, only one study has measured GFAP in cognitively normal subjects followed over time for conversion to dementia (of any kind) [27]. Higher baseline GFAP, measured in serum, was associated to increased risk of dementia, but no significant difference was seen in the change over time of GFAP levels between the cognitively normal and dementia groups [27]. No studies have been done in patients with mild cognitive impairment (MCI) investigating whether plasma GFAP can predict future conversion to AD dementia, specifically.

In this study, our aim was to evaluate plasma GFAP as a potential plasma biomarker of AD in MCI patients, and assess its association with AD-related Aβ pathology and conversion to AD dementia. Early identification of patients that are more likely to have worse cognitive outcome could positively affect their clinical management and help fast-track the diagnostic process; moreover, these patients could be selected for inclusion in clinical trials with disease-modifying drugs currently under development. The study included 160 subjects with a baseline clinical diagnosis of MCI, followed for an average of 4.7 years. CSF and plasma samples were collected at baseline and follow-up. Patients were divided according to clinical diagnosis into the groups *stable MCI* (those who did not progress to AD dementia or other dementias), *MCI-AD* (those who progressed to dementia due to AD), and *MCI-other* (those who progressed to dementia due to other non-AD diseases). Each diagnostic groups was further stratified in Aβ-positive and Aβ-negative based on CSF Aβ42/40 and Aβ42/total tau (T-tau) ratios.

## Materials and methods

### MCI clinical cohort

The present study includes 160 patients referred to the Memory Clinic at Skåne University Hospital, Malmö. CSF data from the cohort have previously been published [28]. At the clinical baseline visit, physicians with an expertise in cognitive disorders performed a thorough physical, neurological, and psychiatric examination, as well as a clinical interview focusing on cognitive symptoms and ADL function. Furthermore, analysis of *APOE* genotype was performed.

Patients with MCI at baseline had to fulfill the criteria by Petersen, including (1) memory complaint, preferably corroborated by an informant; (2) objective memory impairment adjusted for age and education, as judged by the physician; (3) preservation of general cognitive functioning, as determined by the clinician’s judgment based on a structured interview with the patient and a Mini Mental Status Examination (MMSE) score greater than or equal to 24; (4) zero or minimal impairment of daily life activities; and (5) not fulfilling the DSM-IIIR criteria for dementia [29]. Patients with other causes of cognitive impairment, including subdural hematoma, brain tumor, CNS infection, schizophrenia, major depressive episode, and current alcohol abuse were excluded. However, MCI subjects were allowed to show signs of white matter changes or silent brain infarcts, because these changes are frequent in elderly subjects with or without cognitive deficits. Similarly, MCI patients with mild to moderate depressive symptoms and low plasma concentrations of vitamin B12 or folate were not excluded. The included patients with MCI at baseline subsequently developed a certain type of dementia or remained cognitively stable for an average of 4.7 years. The patients with MCI who received a diagnosis of AD during clinical follow-up were required to meet the DSM-IIIR criteria for dementia and the criteria of probable AD defined by NINCDS-ADRDA [30]. Subjects who during follow-up were diagnosed as having vascular dementia (VaD) fulfilled the DSM-IIIR criteria of dementia and the requirements for probable VaD by NINDS-AIREN [31]. The consensus criteria by McKeith and coworkers were applied when diagnosing dementia with Lewy bodies (DLB) [32]. Subjects with progressive supranuclear palsy (PSP) fulfilled the criteria by Litvan et al. [33] and Höglinger et al. [34]. The clinical diagnoses of all patients were reviewed by a consensus group consisting of three medical doctors with special interest in cognitive disorders. The study was approved by the Ethics Committee at the University of Lund and the patients and/or their relatives gave their informed consent (for research). Patients were divided according to clinical diagnosis into the groups *stable MCI* (those who did not progress to AD dementia or other dementias, *n* = 79), *MCI-AD* (those who progressed to dementia due to AD, *n* = 47), and *MCI-other* (those who progressed to dementia due to other diseases: *n* = 34, of which VaD = 25, DLB = 4, PSP = 3, other neurological diseases = 2).

### Plasma and CSF sampling

Blood and CSF samples were collected in the morning during the same visit, with participants non-fasting. Blood was collected in six EDTA-plasma tubes and centrifuged (2000*g*, + 4 °C) for 10 min. Following centrifugation, plasma was aliquoted into 1.5-ml polypropylene tubes (1 ml plasma in each tube) and stored at − 80 °C within 30–60 min of collection. CSF was collected by lumbar puncture and stored at − 80 °C in polypropylene tubes following the Alzheimer’s Association flow chart for lumbar puncture and CSF sample processing [35].

### Plasma and CSF analysis

Plasma GFAP was measured with Simoa GFAP Discovery kits for SR-X (Quanterix®, Billerica, MA, USA) according to the manufacturer’s instructions. The levels of CSF total tau (T-tau) and tau phosphorylated at Thr181 (P-tau181) were determined using xMAP technology as previously described [36]. CSF Aβ40 and Aβ42 levels were analyzed by electrochemiluminescence technology (Meso Scale Discovery, Gaithersburg, Maryland, USA), using the MS6000 Human Abeta 3-Plex Ultra-Sensitive Kit, following the manufacturer’s recommendation. An Aβ-positive subject was defined as having a CSF Aβ42/40 value below 0.07. The cut-off was previously calculated by Youden index as the best to separate AD patients from cognitively healthy controls [28]. Groups were also divided in CSF Aβ42/T-tau-positive and Aβ42/T-tau-negative groups using a cut-off previously calculated in the same cohort using Youden index [28]. A cut-off of 7.3 was the best to separate AD patients from healthy controls.

### Statistical analysis

Analyses were performed using SPSS v.24 (IBM) and R v.3.5.3. Data were visualized using either Prism 8.4 (Graphpad) or R. Group differences were assessed in univariate general linear models, adjusted for age and sex and post hoc least significant difference (LSD) tests for pairwise group comparisons. Biomarkers values were LOG10 transformed prior to this analysis. Diagnostic accuracies were assessed with receiver operating characteristic (ROC) curve analysis and binary logistic regression models. Akaike information criterion (AIC) was calculated for each logistic regression model. Sensitivities and specificities for the biomarkers were calculated at Youden index thresholds. Linear mixed-effect model with random intercept adjusted for age and sex was used to determine biomarker slopes over time and differences between groups. Spearman *r* was used to measure correlations between biomarkers. *P* values < 0.05 were considered significant.

## Results

Group sizes, age, and gender distribution for subgroups are shown in Table 1. Of these 160 patients with MCI, 159 had GFAP measurements at baseline and one case had only the follow-up measurement of GFAP. One hundred and ten subjects had both baseline and follow-up measurements. The average length of the follow-up was 4.7 years.

**Table 1:** Demographic and clinical data

	Stable MCI (Aβ−)	Stable MCI (Aβ+)	MCI-AD (Aβ+)	MCI-other (Aβ−)	MCI-other (Aβ+)
**Characteristics**					
*N*	58	21	47	25	9
Age	69 (8)	69 (6)	76 (7)	73 (7)	74 (6)
Gender (% female)	55%	48%	75%	44%	33%
*APOE* genotype (% 1 or 2 ε4 alleles)	29%	81%	81%	44%	78%
**CSF**					
Aβ42/40	0.10 (0.02)	0.05 (0.01)	0.05 (0.01)	0.10 (0.02)	0.06 (0.01)
T-tau (pg/mL)	68 (29)	111 (72)	148 (70)	90 (43)	75 (33)
P-tau181 (pg/mL)	26 (11)	40 (21)	54 (22)	30 (12)	33 (11)
**Plasma**					
GFAP baseline (pg/mL)	36 (17)	46 (25)	67 (24)	42 (16)	52 (11)
GFAP follow-up (pg/mL)	43 (16)	65 (41)	94 (34)	67 (27)	78 (34)

Values are expressed as mean (SD). *Abbreviations*: *Aβ* β-amyloid, *Aβ+* Aβ positive, *Aβ*− Aβ negative, *CSF* cerebrospinal fluid, *GFAP* glial fibrillary acidic protein

GFAP baseline values in Aβ-negative MCI subjects correlated significantly with age *(r* = 0.57, *p* < 0.0001) and CSF Aβ42/40 (*r* = − 0.33, *p* = 0.003) (Table 2). In Aβ-positive MCI patients, GFAP also correlated with CSF T-tau (*r* = 0.29, *p* = 0.01) and CSF P-tau181 (*r* = 0.28, *p* = 0.01) (Table 2).

**Table 2:** Correlations between plasma GFAP baseline levels and age, CSF Aβ42/40, CSF T-tau, and CSF P-tau181

		Age	Aβ42/40	T-tau	P-tau181
**Aβ−**	*r*	0.57	− 0.33	0.06	− 0.03
	*p*	< 0.0001	0.003	0.61	0.79
**Aβ+**	*r*	0.46	−0.18	0.29	0.28
	*p*	< 0.0001	0.13	0.01	0.01

*r* Spearman’s r, *Aβ+* Aβ positive, *Aβ*− Aβ negative

### Plasma GFAP for detection of AD pathology

**Fig. 1:**
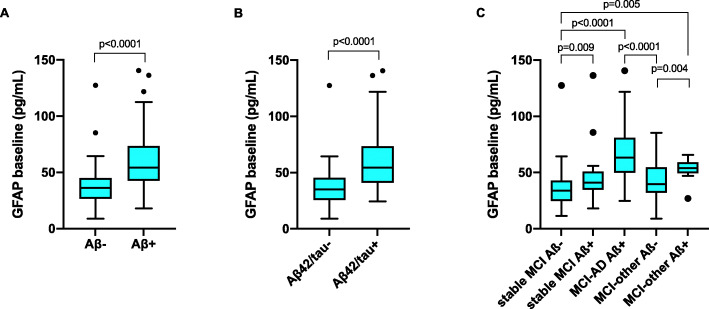
**a** Plasma GFAP in the Aβ-positive (Aβ+, CSF Aβ42/Aβ40 < 0.07) and Aβ-negative (Aβ−, CSF Aβ42/Aβ40 > 0.07) groups. **b** Plasma GFAP in the Aβ42/tau positive (Aβ42/T-tau+, CSF Aβ42/T-tau < 7.3) and Aβ42/tau negative (Aβ42/tau−, CSF Aβ42/T-tau > 7.3) groups. **c** Plasma GFAP in the stable MCI, MCI-AD and MCI-other groups stratified by Aβ status. Line across represents median, box represents interquartile range (IQR), bars represent min and max value (within ± 1.5 IQR). *P* values were calculated with univariate linear model and least significant differences (LSD) post hoc tests, with adjustments for age and sex

We first analyzed the whole cohort comparing plasma GFAP in groups defined according to their CSF Aβ42/40 and Aβ42/T-tau status. Plasma GFAP levels at baseline were significantly different between CSF Aβ42/40-positive and CSFAβ42/40-negative groups as well as between CSF Aβ42/T-tau-positive and CSF Aβ42/T-tau-negative groups (both *p* < 0.0001) (Fig. 1a, b). We then compared those patients who progressed to dementia due to AD (MCI-AD) to those who remained stable (stable MCI) and those who progressed to dementia due to other diseases (MCI-other). The last two groups were subdivided in Aβ-positive or Aβ-negative according to the presence or absence of biomarker evidence of brain amyloidosis based on CSF Aβ42/40. The MCI-AD group had significantly higher plasma GFAP concentrations than Aβ-negative cognitively stable MCI and Aβ-negative MCI-other (both *p* < 0.0001) (Fig. 1c). Aβ-positive stable MCI and Aβ-positive MCI-other had significantly higher concentration of plasma GFAP compared to stable MCI Aβ-negative cases (*p* = 0.01 both) (Fig. 1c). The Aβ-positive MCI-other group had significantly higher GFAP levels than the Aβ-negative MCI-other group (*p* < 0.004) (Fig. 1c). No significant differences were present between the different Aβ-positive diagnostic groups or the different Aβ-negative diagnostic groups.

**Fig. 2:**
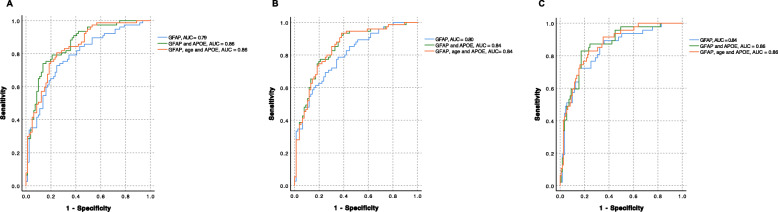
ROC curves for plasma GFAP and GFAP combined with additional predictors (APOE, age) to assess accuracy when predicting Aβ positivity (**a**), Aβ42/T-tau positivity (**b**), and MCI-AD status (**c**). AUC, area under the curve

Next plasma GFAP baseline measurements were tested alone or when combined with age and/or *APOE* ε4 status when predicting the CSF Aβ status. Receiver operating characteristics (ROC) curve analysis showed the greatest area under the curve (AUC) for GFAP combined with *APOE* ε4 status (AUC = 0.86) (Fig. 2a, Table 3). This combination had a significantly higher AUC than GFAP by itself (*p* = 0.02). Adding age to the model with GFAP and *APOE* ε4 status did not further improve the accuracy (AUC = 0.86) (Table 3). Binary logistic regression with Aβ-positive status as outcome showed that plasma GFAP combined with *APOE* ε4 status was the best predictor, with the lowest Akaike information criterion (AIC, 152; Δ AIC = − 24) (Table 2).

**Table 3:** Combined results from binary logistic regression models and ROC curve analysis for prediction of Aβ-positive status

	*p*	AIC	AUC	95% CI	Difference AUC (*p*)	Δ AIC with GFAP
GFAP	< 0.0001	177	0.79	0.72–0.86	–	–
Age	< 0.0001	208	0.64	0.56–0.73	–	–
*APOE* ε4 status	< 0.0001	187	0.73	0.65–0.81	–	–
GFAP and age	< 0.0001	179	0.78	0.71–0.86	ns	2
GFAP and *APOE* ε4 status	< 0.0001	152	0.86	0.80–0.92	0.02	−25
GFAP, age, and *APOE* ε4 status	< 0.0001	153	0.86	0.80–0.92	0.02	−24

Aβ-positive status was defined as CSF Aβ42/40 < 0.07. *AIC* Akaike information criterion, *AUC* area under the curve, *95% CI* 95% confidence intervals, *difference AUC (p)* difference between AUCs measured with DeLong test (AUC of GFAP used as reference), *Δ AIC* difference in AIC (AIC of GFAP used as reference), *ns* not significant

The same approach was used for the identification of CSF Aβ42/T-tau-positive status. Plasma GFAP combined with *APOE* e4 status (with or without age) had the greatest AUC (0.84 for both) (Fig. 2b, Table 4). GFAP alone had the same AUC as in combination with age (0.80) (Fig. 2b, Table 4). Binary logistic regression with Aβ42/T-tau-positive status as outcome showed that GFAP combined with *APOE* was the best predictor, with the lowest AIC (157, Δ AIC = − 15) (Table 4). AUCs for combinations of GFAP, *APOE* ε*4*, and age were not significantly superior to the AUC of GFAP by itself (differences between AUCs not significant).

**Table 4:** Combined results from binary logistic regression models and ROC curve analysis for prediction of Aβ42/T-tau-positive status

	*p*	AIC	AUC	95% CI	Δ AIC with GFAP
GFAP	< 0.0001	172	0.80	0.73–0.87	–
Age	< 0.0001	209	0.64	0.55–0.73	–
*APOE* ε4 status	< 0.0001	196	0.68	0.59–0.76	–
GFAP and age	< 0.0001	174	0.80	0.73–0.87	2
GFAP and *APOE* ε4 status	< 0.0001	157	0.84	0.78–0.91	−15
GFAP, age, and *APOE* ε4 status	< 0.0001	159	0.84	0.78–0.91	−13

Aβ42/T-tau-positive status was defined as CSF Aβ42/T-tau < 7.3. *AIC* Akaike information criterion, *AUC* area under the curve, *95% CI* 95% confidence intervals, *Δ AIC* difference in AIC (AIC of GFAP used as reference)

GFAP cut-off for Aβ-positive (> 44.7 pg/mL) had a sensitivity and of 73% and 75%, respectively. GFAP cut-off for Aβ42/T-tau-positive (> 44.9 pg/mL) had a sensitivity of 69% and a specificity of 75%.

### Plasma GFAP can predict subsequent of conversion to AD dementia

Plasma GFAP baseline measurements were tested alone or combined with age and/or presence of at least one *APOE* ε*4* allele for accuracy in prediction of conversion to AD dementia at follow-up (MCI-AD). ROC curve analysis showed the greatest AUC for plasma GFAP combined with *APOE* (AUC = 0.86) (Fig. 2c, Table 5). Adding age to the model did not improve the accuracy (AUC = 0.86). Binary logistic regression with MCI-AD status as outcome showed GFAP combined with *APOE* ε*4* and age as the best predictor having the lowest AIC (136, Δ AIC = − 12) (Table 5). AUCs for combinations of GFAP, *APOE* ε*4* and age were not significantly superior to the AUC of GFAP by itself (differences between AUCs not significant).

**Table 5:** Combined results from binary logistic regression models and ROC curve analysis for prediction of subsequent development of AD dementia (MCI-AD)

	*p*	AIC	AUC	95% CI	Δ AIC with GFAP
GFAP	< 0.0001	148	0.84	0.77–0.91	–
Age	< 0.0001	171	0.73	0.64–0.81	–
*APOE* ε4 status	< 0.0001	177	0.67	0.58–0.76	–
GFAP and age	< 0.0001	145	0.83	0.76–0.90	−3
GFAP and *APOE* ε4 status	< 0.0001	140	0.86	0.80–0.93	−8
GFAP, age, and *APOE* ε4 status	< 0.0001	136	0.86	0.81–0.92	−12

*AIC* Akaike information criterion, *AUC* area under the curve, *95% CI* 95% confidence intervals, *Δ AIC* difference in AIC (AIC of GFAP used as reference)

GFAP cut-off for an MCI-AD status (> 54.1 pg/mL) had a sensitivity and specificity of 72% and 85%, respectively.

### Longitudinal changes in plasma GFAP

**Fig. 3:**
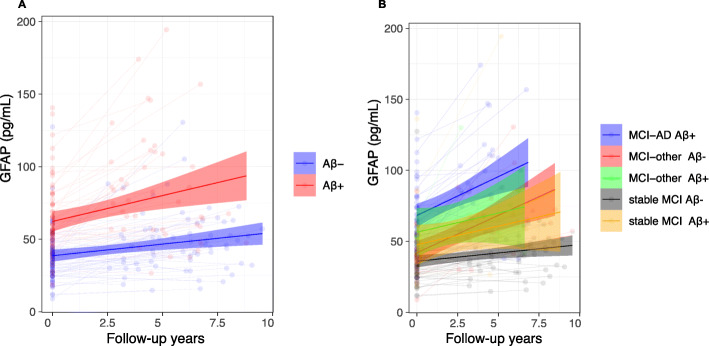
Longitudinal slopes for plasma GFAP from linear mixed-effects model adjusted for age and sex. *X*-axis represents time between baseline and follow-up plasma sampling; *y*-axis represents plasma concentrations in pg/mL. Plasma GFAP measurements were stratified by Aβ-positive (Aβ+) status defined by CSF Aβ 42/40 < 0.07 (**a**). Slopes for Aβ-positive subjects were significantly steeper than Aβ-negative (*p* = 0.007). In **b**, plasma GFAP measurements were stratified by clinical diagnosis at follow-up and Aβ status. GFAP slopes in MCI-AD and Aβ-negative MCI-other subjects were significantly steeper compared to stable MCI Aβ-negative (*p* < 0.0001 both) and stable MCI Aβ-positive (*p* = 0.049, *p* = 0.037, respectively)

Slopes for plasma GFAP adjusted for age and gender showed a significant longitudinal increase in Aβ-negative (β = 2.02, *p* < 0.0001), with a larger increase in the Aβ-positive group compared to Aβ-negative (*β* = 2.06, *p* = 0.01 compared to Aβ-negative) (Fig. 3a).

When looking at changes over time for different cognitive groups, plasma GFAP showed a significantly higher longitudinal increase in MCI-AD and Aβ-negative MCI-other compared to Aβ-negative stable MCI (*p* < 0.0001 both) and Aβ-positive stable MCI (*p* = 0.049 and *p* = 0.034, respectively) (Fig. 3b). No significant difference was seen between Aβ-positive MCI-other and stable MCI groups and MCI-AD and MCI-other groups.

## Discussion

In this study, we assessed the potential of plasma GFAP as a diagnostic and prognostic biomarker for AD in a longitudinal MCI cohort. We observed that high GFAP concentration measured at baseline was a relatively strong indicator of AD pathology and could accurately predict future development of AD dementia. Differences at baseline seemed to be associated with (and possibly driven by) the Aβ status; higher concentrations at baseline were in fact observed in every Aβ-positive subgroup compared to Aβ-negative ones. When looking at AUCs as measures of accuracy, the presence of at least one *APOE* ε4 allele moderately increased the accuracy of GFAP in detecting AD pathology, but not the accuracy in predicting conversion to AD dementia (no significant difference between AUCs), although the model was overall improved (Δ AIC = − 8). This suggests that plasma GFAP is an accurate biomarker for AD diagnosis and progression.

All cognitive groups showed a mild to moderate increasing trend over time for plasma GFAP (Fig. 3b). However, the MCI-AD and Aβ-negative MCI-other group showed a significantly steeper trajectory compared to the groups that remained cognitively stable. It is possible that GFAP generally increases over time because of its significant correlation with age in both Aβ-negative and Aβ-positive groups (Aβ-: r = 0.57; Aβ+: *r* = 0.46); however, this could also suggest that higher concentrations of plasma GFAP are associated to general worsening in clinical symptoms, as the steepness of the increase in GFAP was greater in all groups that evolved to dementia. The lack of significance in the difference between the Aβ-positive MCI-other group compared to the stable MCI ones is probably due to the small size of the first group (*n* = 9). This hypothesis is also reinforced by recent studies on CSF and plasma that show an inverse correlation between concentrations of GFAP and cognition [22, 27, 37]. However, one study from Verberk and colleagues [27], following cognitively normal subjects over time for evolution to dementia (AD and non-AD combined), showed that the dementia group had no steeper trend of increase over time compared to the cognitively healthy group.

There are a series of considerations to take into account when comparing the two studies. The studies have comparable follow-up times (average 4.7 years versus median 3.6 years) and sample sizes of patients with follow-up measurements (160 versus 92 subjects). Both studies also show that patients later evolving to dementia have higher baseline GFAP concentrations. However, in Verberk et al., only 9% of patients in the longitudinal cohort developed dementia (six AD dementia, one progressive supranuclear palsy, one primary progressive aphasia), probably also due to the relatively young age of the population (average 61 years) and low percentage (20%) of CSF Aβ-positive subjects. This might have affected the power of the statistical analysis when comparing the longitudinal slopes between the dementia and cognitively healthy groups. It should also be considered that, in Verberk et al., GFAP was measured in serum, as opposed to plasma in the present study. So far, only one study has compared plasma and serum concentrations of GFAP in a traumatic brain injury cohort, with good correlation but significantly higher concentrations of GFAP in plasma [19]. Extensive head-to-head comparisons are yet to be done between the two different methods of analysis and it cannot be excluded that the presence of clotting agents in the sample might affect the results.

In our study, the finding of a strong association with Aβ is not surprising, given the fact that AD (which is defined by Aβ deposition in plaques) is associated with astrogliosis and release of GFAP from astrocytes [3]. However, the results from previous studies with amyloid-PET imaging suggest that as the Aβ load increases in brain along the course of disease, astrogliosis decreases [9, 11]. A similar dynamic association was observed in a recent cross-sectional study on plasma GFAP, where linear positive associations between brain Aβ load measured with amyloid-PET were observed in subjects at earlier stages of disease and diverged in more severe disease stages [25]. Our cohort had an adequate follow-up time (4.7 years on average); however, we cannot exclude that at a longer follow-up a similar association could be observed. Although GFAP is not specific for AD, the high accuracy showed in detecting AD pathology and conversion to AD dementia suggest that plasma GFAP could be a useful indicator of the astroglial activation component of the multifaceted pathology in AD. In our cohort, plasma GFAP also showed a significant negative correlation to CSF Aβ42/40 in Aβ-negative subjects (Table 2), indicating that lower values of CSF Aβ42/40 (still in the normal range) are associated with higher plasma GFAP. This also suggests that plasma GFAP could be very early marker in the pre-dementia phase, like that of our patients at baseline. The strong association with Aβ status also suggests that plasma GFAP could add to and complement the information from plasma Aβ, as immunoassay measurements of Aβ42 and Aβ40 (and their ratio) still require further optimization [38–40].

### Limitations

One limitation of our study was the lack of a cognitively healthy control group. However, we did comparisons between the AD group and the stable MCI Aβ-negative, which had a non-progressing type of cognitive impairment and no objectifiable evidence of underlying neurodegenerative disease. Another limitation is the lack of PET imaging data for determination of Aβ status; we have, however, defined Aβ-positive patients based on the CSF Aβ42/40 ratio, which has consistently shown a strong association with brain Aβ load evaluated by PET or at neuropathology [41, 42].

## Conclusions

In conclusion, our results show that plasma GFAP is associated to AD-type pathology and can accurately predict clinical progression to AD dementia, making it a potential candidate to add to the blood-based biomarker panel for AD.

## Data Availability

Anonymized data will be shared by request from a qualified academic investigator for the sole purpose of replicating procedures and results presented in the article and as long as data transfer is in agreement with EU legislation on the general data protection regulation and decisions by the Ethical Review Board of Sweden and Region Skåne, which should be regulated in a material transfer agreement.

## Abbreviations

Aβ: Amyloid β
AD: Alzheimer’s disease
AIC: Akaike information criterion
AUC: Area under the curve
CBS: Corticobasal syndrome
CSF: Cerebrospinal fluid
DLB: Lewy body dementia
MCI: Mild cognitive impairment
PSP: Progressive supranuclear palsy
P-tau181: Phosphorylated tau
ROC: Receiver operating characteristic
Simoa: Single molecule array
T-tau: Total tau
VaD: Vascular dementia
